# Relationship between Response to PDE5 Inhibitors and Penile Duplex Doppler Ultrasound in Erectile Dysfunction

**DOI:** 10.3390/medsci6020028

**Published:** 2018-03-26

**Authors:** Ercan Ogreden, Ural Oğuz, Erhan Demirelli, Alptekin Tosun, Orhan Yalçın

**Affiliations:** 1 Department of Urology, Giresun University Faculty of Medicine, Giresun, Sağlık Uygulama ve Araştırma Hastanesi Nizamiye Mah. Mumcular Sok. No: 1/1, 1 nolu Sağlık Ocağı Arkası, Merkez, Giresun P.K. 28200, Turkey; ural.oguz@giresun.edu.tr (U.O.); erhan.demirelli@giresun.edu.tr (E.D.); oyalcin56@yahoo.com (O.Y.); 2 Department of Radiology, Giresun University Faculty of Medicine, Giresun P.K. 28200, Turkey; alptekin.tosun@giresun.edu.tr

**Keywords:** comorbidities, erectile dysfunction, penile duplex Doppler ultrasound, penile pathology, phosphodiesterase type-5 inhibitors

## Abstract

The relationship between the results of penile duplex Doppler ultrasound (PDDU) and response to vardenafil was investigated in patients diagnosed with erectile dysfunction (ED). Data from 148 patients with ED were analyzed retrospectively. Patients who did not respond to therapy were classified in to Group I (*n* = 32), those who responded partially were classified into Group II (*n* = 40), and complete responders were classified into Group III (*n* = 76). Age, comorbidities, and vascular and penile pathologies were compared among the three groups. While diabetes mellitus (DM) and dyslipidemia positivity adversely affected the response to treatment, the presence of hypertension (HT), Peyronie’s disease and priapism increased the therapeutic response to the treatment (*p* < 0.05). Arterial insufficiency was present in 20 (30.3%), 25 (37.9%) and 21 (31.8%) of the patients in Group I, Group II and Group III, respectively (*p =* 0.001). Venous insufficiency was observed in three (14.3%) patients in Group I and in eight (85.7%) patients in Group III (*p =* 0.001). Arterial/venous insufficiency was seen in 9 (30%), 14 (46.7%) and 7 (23.3%) of the patients in Group I, Group II and Group III, respectively (*p =* 0.001). The response rate to treatment was highest in normal patients according to PDDU, followed by patients with venous insufficiency. In addition, it was found that DM decreased the response to treatment, whereas the response increased in cases with HT, priapism and Peyronie’s disease.

## 1. Introduction

Erectile dysfunction (ED) is defined as the inability to achieve and maintain an erection sufficient for satisfactory sexual intercourse [1]. Organic and psychogenic factors are responsible for the pathogenesis of ED. Because the penis has an important vascular bed, vascular pathologies are more frequent. Vascular pathologies may be arteriogenic, venogenic and/or of a mixed type [2]. In vasculogenic ED; advanced age, diabetes mellitus (DM), hypertension (HT), dyslipidemia, and atherosclerosis are the main risk factors. Penile pathologies, such as priapism and Peyronie’s disease, can also cause ED [3,4]. The basic mechanisms involved in vasculogenic ED pathophysiologies are a reduction in nitric oxide (NO) synthesis due to bioavailability. Furthermore, the interaction between NO and superoxide anions causes an increase in free oxygen radicals. Free oxygen radicals in turn lead to neuronal and endothelial damage. Endothelial damage is associated with endothelial dysfunction and atherosclerosis [5]. Phosphodiesterase type-5 inhibitors (PDE5Is, sildenafil, tadalafil, udenafil, vardenafil, avenafil, etc.) are used in current treatments of patients with vasculogenic ED [6]. However, diagnostic tests are recommended in patients who do not benefit from oral agents or when surgical treatment is planned. The most important of these tests is penile duplex doppler ultrasonography (PDDU). In this imaging technique, a careful evaluation is performed following an intracavenousal injection. This acts to ensure a functional penile erection, and so, reliable information about penile arterial and venous system [7]. In this study, we aimed to investigate the relationship between PDDU results and the response to vardenafil in patients diagnosed with ED.

## 2. Materials and Methods

Data from 260 patients admitted to our clinic with a history of ED that persisted for longer than 6 months, between May 2009 and April 2015, were evaluated retrospectively. Sexual history, physical examination findings, International Index of Erectile Function-5 (IIEF-5) scores, systemic diseases that are potential risk factors for ED, and PDDU data were obtained from the patients’ classic and electronic files. Out of all patients who underwent PDDU, those with a history of major pelvic surgery such as radical prostatectomy, neurologic diseases such as degenerative neuropathies, and those with a history of pelvic or perineal trauma were excluded from the evaluation. Patients who were diagnosed with priapism and who were treated after 4 h were excluded from the study. Data from 148 patients who had severe ED according to the IIEF-5 score were included in the study. The serum testosterone (>346 ng/dL), luteinizing hormone (LH), follicle stimulating hormone (FSH) and prolactin (<25 µg/L) levels of the patients included in the study were normal. PDDU, which is standard in our clinic, was performed following a 50 mg intracavernosal injection of papaverine. Before the procedure, information about the possible complications of the disease was given, and signed consent forms were obtained from the patients. Following tourniquet and tactile stimulation, the procedure was performed on the erect penis using ultrasound equipment with a superficial 12 MHz probe. Five-, ten-, fifteen-, and thirty-minute waveforms were obtained from the cavernosal artery using the penoscrotal angle approach; peak systolic velocity (PSV), end diastolic velocity (EDV), and resistance index (RI) values were recorded. PSV values above 30 cm/s and RI values above 0.80 were considered to be a normal vascular response. PSV is the best Doppler indicator of arteriogenic ED. If PSV values were below 30 cm/s and RI values were below 0.80, they were considered as indicators ofarterial insufficiency. EDV is the best Doppler indicator of venogenic ED. PSV values above 30 cm/s and RI values below 0.80 were considered as venous insufficiency. Patients were started on vardenafil 10 mg orodispersible tablets (ODT), a PDE5Is. The ODT form of vardenafil is preferred because its oral absorption is not affected by stomach acid. A biweekly dosage adjustment was done orally, and patients were questioned following 12 weeks of treatment. In order to assess the response to treatment, an IIEF-5 questionnaire was used and ED was classified into five categories based on the scores: severe (5–7), moderate (8–11), mild to moderate (12–16), mild (17–21), and no ED (22–25). The IIEF-5 score following the vardenafil ODT treatment was classified as: a score within the severe ED range was classified as the non-response group (Group I (*n* = 32)), a score within moderate ED range was classified as the partial response group (Group II (*n* = 40)) and those who were within mild ED or normal erectile function range were classified as the complete response group (Group III (*n* = 76)). The distribution of age, comorbidities, such as DM, HT and dyslipidemia, type of vascular pathology (arterial, venous, arterial and venous insufficiency) if present, and the relationship between priapism and Peyronie’s disease were compared among the groups.

This study was approved by the ethical committee of Giresun University Faculty of Medicine. (approval No. KAEK-01).

### Statistical Analysis

Data obtained in this study were analyzed with theSPSS 20 (IBM SPSS Statistics; Armonk, NY, USA) package program. Because of unit numbers, the Shapiro–Wilks test was conducted to check whether the variables came from a normally distributed population. When the differences among the groups were examined, the Kruskal–Wallis-H test was used in cases where variables did not come from a normal distribution. If the Kruskal–Wallis-H test revealed significant differences, those groups that contained the differences were detected using the Post-Hoc Multiple Comparison Test. The Chi-squared test was used when the relationships of nominal variables were analyzed among the groups. Pearson’s Chi-squared test was performed. Statistical significance was accepted when *p* < 0.05.

## 3. Results

The average age of the patients was 56.3 (27–80 years). The average age was 57.7 (45–65 years) in Group I, 57.9 (33–80 years) in Group II, and 54.9 (27–79 years) in Group III (*p =* 0.404) (Table 1).

DM was present in 30 (93.7%) patients in Group I, in 38 (95%) patients in Group II, and in 32 (42.1%) patients in Group III. It was found that the distribution of patients with no DM was significant in the complete response group (Group III) (*p =* 0.001). HT was observed in 16 (50%) patients in Group I, in 27 (67.5%) patients in Group II, and in 67 (88.1%) patients in Group III. It was found that the distribution of patients with HT was significant in Group III (*p =* 0.001). Dyslipidemia was found in 26 (81.2%) patients in Group I, in 39 (97.5%) patients in Group II, and in 45 (59.2%) patients in Group III. It was found that the distribution of patients with dyslipidemia was significant in Group III (*p =* 0.001).

According to the results of PDDU, priapism was detected in 22 (14.8%) of the 148 patients. All the patients with priapism were found in Group III. There was no priapism in any patients in Group I and II (*p =* 0.001). It was determined that erectile dysfunction did not develop in patients who underwent intervention due to priapism within the first 4 hours. Peyronie’s disease was present in 16 (50%) patients in Group I, in 9 (22.5%) patients in Group II, and in 18 (23.6%) patients in Group III (*p =* 0.013). The response to the vardenafil treatment in Peyronie’s patients was significantly higher (Table 2).

According to the results of PDDU; in Group I, the number of patients with arterial insufficiency was 20 (30.3%), the number of patients with venous insufficiency was three (14.3%), and the number of patients with arterial and venous insufficiency was nine (30%). However, there were not any normal patients in this group (*p =* 0.001). In Group II, the number of patients with arterial insufficiency was 25 (62.5%), the number of patients with both arterial and venous insufficiency was 14 (35%), and the number of normal patients was one (3.2%) (*p =* 0.001). The number of patients with arterial insufficiency was 21 (27.6%) in Group III, while number of patients with venous insufficiency was 18 (23.7%). The number of patients with both arterial and venous insufficiency was seven (9.2%) in this group, whereas the number of patients with normal results was 30 (39.5%). After reviewing these data, it was found that the rate of patients with venous insufficiency, as well as with normal results, was statistically significantly higher in Group III than in Group I and Group II (*p =* 0.001). The number of patients with PSV >30 cm/s was three (5.6%) in Group I, one (1.9%) in Group II, and 50 (92.6%) in Group III. The number of the patients with PSV <30 cm/s was 29 (30.9%) patients in Group I, 39 (41.5%) patients in Group II, and 26 (27.7%) patients in Group III (*p =* 0.001). The number of patients with EDV >8 cm/s was 12 (19%), 14 (22.2%), and 37 (58.7%) patients in Group I, Group II, and Group III, respectively. The number of patients with EDV <8 cm/s was 20 (23.5%) patients in Group I, 26 (30.6%) patients in Group II, and 39 (45.9%) patients in Group III (*p =* 0.296). The number of the patients with RI >0.8 was 14 (19.7%) patients in Group I, 11 (15.5%) patients in Group II, and 46 (64.8%) patients in Group III. The number of patients with RI <0.8 was 18 (23.4%), 29 (37.7%), and 30 (39%) patients in Group I, Group II and Group III, respectively (*p =* 0.003) (Table 3).

## 4. Discussion

ED is the most common sexual disorder in men. For normal erectile function, psychosocial, hormonal, neurological, vascular, and cavernosal factors coordinate; defects in one or more of these factors lead to ED. In ED treatment, PDE5Is are the gold standard. Current selective PDE5Is include sildenafil, tadalafil, udenafil, vardenafil, and avanafil. PDE5Is are also used in the treatment of ED secondary to aging, DM, HT, dyslipidemia, and atherosclerosis, as well as in general the population [8,9]. These etiologic factors cause impairment in the structure of the corpus cavernosum and result in loss of erectile function.

ED is a worldwide health problem and its incidence increases with age. In developed countries, improved life expectancy also increases the general incidence of ED among the general population. Recently obtained epidemiological data have shown that the worldwide prevalence and incidence of ED are 69.2% and 34.5%, respectively [10]. With the increasing age, atherosclerosis which occurs in vascular structures can also affect penile vascular tissue and may cause ED. Atherosclerosis impairs the blood flow in the cavernosal arteries and leads to an accumulation of free oxygen radicals) in the tissue [11]. In our study, the rate of patients with arterial insufficiency was higher in the non-response group when compared with the partial or complete response groups.

ED is three times more common in diabetic patients than in the normal population. In diabetic patients, impairment in NO synthesis, increased free oxygen radicals, oxidative stress-related damage, and increased glycosylation end-product levels are the most important factors leading to ED. It was shown in several studies that the duration and severity of risk factors increase the injury to vascular structures [11,12,13]. The relationship between the duration of diabetes and severity of ED is especially well established. Poor metabolic control in diabetes shortens the time between the onset of diabetes and the development of ED [13]. Arterial structures are affected earlier when compared to trabecular smooth-muscle structures and the venous system. The duration of the diabetes, poor metabolic control, and on-going atherosclerosis cause atrophy in the penile smooth-muscle structures, loss of myofilament, shrinking in cell size, an increase in collagen fibers, a decrease in “gap junction” structures, impairment in ability to relax the trabecular smooth-muscle structures, and venous leakage [14]. This, in turn, hampers the treatment of ED associated with DM. In a retrospective study conducted by Blonde [14], the rate of the patients who responded to treatment was reported as 62%; they concluded that treatment with PDE5Is was effective in achieving and maintaining erectile function (*p* < 0.0001). In our study, it was calculated that the rate of patients diagnosed with DM who responded partially or completely to treatment with PDE5Is was 70%. We believe that this difference could be related to the fact that the dosage was not standard, and a different preparation of PDE5Is was used in Blonde’s study. In vitro studies report that vardenafil is 10 times more potent than sildenafil [15]. This study too shows that different active substances could have different effects. In a study comparing various doses of sildenafil, 100 mg sildenafil was shown to be more effective [16]. The lack of dose standardization might have reduced the total effect. Additionally, in the development of ED in diabetic cases, an accompanying neurologic component, along with the vascular pathology, contributes to a decrease in the response to treatment. In cases where no response is obtained with vardenafil 10 mg, an increase in dosage and an evaluation of the patient in terms of peripheral neuropathy could be beneficial.

HT is considered to be a major risk factor for ED. ED is present in approximately 30% of hypertensive patients. The mutual mechanism between HT and ED is endothelial dysfunction. In patients with HT, due to mechanical damage and increased oxidative stress, deterioration of mitochondrial and endoplasmic reticulum of the endothelial cells are responsible for the development of ED. ED can develop because of hemodynamic changes due to high blood pressure, and increased atherosclerosis, which can result from antihypertensive treatments [17]. Although HT is a major risk factor for ED, in a meta-analysis, it was shown that compared to placebo, vardenafil provided significant improvements in patients with ED. In the same study it was reported that vardenafil provoked similar effects in patients with or without HT [18].

Dyslipidemia is a well-known risk factor for atherosclerosis. Dyslipidemia leads to occlusion by increasing the lipid accumulation in vascular lesions and accelerating the atherosclerotic process. In addition, endothelial dysfunction is held to be responsible for the main pathology. Atherosclerotic lesions extend through the internal pudendal and cavernosal arteries and decrease penile blood flow. Although statin-group drugs are used as a first-line treatment for dyslipidemia, studies have shown that these types of agents can cause ED. As a result, the treatment of patients with dyslipidemia involves PDE5Is [19]. In our study, the response rates of patients with dyslipidemia to treatment with PDE5Is was 81.2% in Group I, 97.5% in Group II, and 59.2% in Group III, respectively (*p =* 0.001). It was observed that the response to PDE5Is was diminished in patients with dyslipidemia. Additionally, the rates of patients with arterial insufficiency, and both arterial and venous insufficiency, were higher in the group where no response to treatment was achieved compared to the other treatment groups. In the treatment group with prominent venous insufficiency, the response to treatment with PDE5Is was favorable.

Priapism is a rare pathology defined as a painful erection, which lasts for more than 4 h, without any sexual desire or stimulation [20]. The prolonged penile erection that occurs in priarism can potentially cause tissue damage. Priapism usually affects patient populations with low NO bioavailability. In this condition, the mechanisms of erection are entirely normal, but there appears to be a lack of mechanisms which control the returning of the penis to its flaccid state. Therefore, prolonged uncontrolled erections are seen in priapism [21]. Histopathologic studies have shown that tissue damage is dependent on the duration of the erection and irreversible damage occurs after 6 h. [22]. The goal of priapism treatment is to empty anoxic blood, decompressing the corpus cavernosum, and thus, providing perfusion. On the basis of this, alleviating pain, ischemia, necrosis, fibrosis, the formation of penile deformity, and probability of erectile dysfunction are the objectives [23]. In our study, all of the cases with priapism were treated in under 4 h and erectile tissue damage was avoided. Therefore, a complete response to treatment with PDE5Is was achieved in all patients who developed priapism.

Peyronie’s disease, a disorder that plays a role in ED, is an inflammatory process characterized by fibroblast proliferation and fibrous plaque formation in the tunica albuginea. Peyronie’s disease is associated with ED in 20–40% of the cases [24]. Ozturk et al. [25] investigated the effectiveness of sildenafil in 39 patients who had Peyronie’s disease and accompanying ED. They showed that a significant improvement occurred in IIEF-5 in the group that received sildenafil (*p =* 0.028). In our study, a partial or complete response rate to the treatment was achieved in 43 patients with Peyronie’s disease (62.8%). In a study, following treatment with PDE5Is, a complete or partial satisfaction rate was reported in 70.8% of patients. In the same study, 90% of the patients who had venous insufficiency according to PDDU findings, were satisfied with the treatment [26]. In our study, the rate of patients with venous insufficiency in Group III was 85.7%.

It is known that approximately 35% of the cases do not respond to PDE5 inhibitors in the treatment of ED. Major reasons for this failure include DM, and severe neurologic and vascular disorders [27]. In our study, the non-response rate to PDE5Is was 21.6%, a figure lower than that found in the literature. We believe that the reason for this is the exclusion of potential conditions that could lead to neurologic ED.

Mulhall et al. [28] reported that the response rate was 25% in ED patients who had venous insufficiency. This figure was 85.7% in our study. The success rate in ED has an inverse correlation with venous insufficiency. It is reported that response to the treatment decreases in patients with a high degree of insufficiency [29]. We did not grade the degree of venous insufficiency, so this could be considered a limitation of our study.

## 5. Conclusions

In this study, we found that the rate of patients with arterial insufficiency was higher in groups of patients who did not respond to PDE5Is. The response rate to the treatment was highest in normal patients according to PDDU findings, followed by patients with venous insufficiency. Additionally, it was found that DM decreases the response to treatment; however, the response was improved in patients who had HT, priapism and Peyronie’s disease.

## Figures and Tables

**Table 1:** Age distribution of according to groups.

								Kruskal Wallis H Test		
		*n*	Mean	Median	Min	Max	SD	Rank Average	H	*p*
Age	Group I	32	57.75	57	45	65	5.62	79.91	1.811	0.404
	Group II	40	57.9	57.5	33	80	10.39	78.91		
	Group III	76	54.91	55	27	79	12.64	69.9		
	Total	148	56.33	57	27	80	10.91			

SD: Standard deviation.

**Table 2:** Distribution of comorbidities according to the groups.

										Chi-Square Test	
		Group I		Group II		Group III		Total			
		*n*	%	*n*	%	*n*	%	*n*	%	Chi-Square	*p*
Diabetes Mellitus	Positive	30	30	38	38	32	32	100	100	46.232	0.001
	Negative	2	4.2	2	4.2	44	91.7	48	100		
	Total	32	21.6	40	27	76	51.4	148	100		
Hypertension	Positive	16	14.5	27	24.5	67	60.9	110	100	18.519	0.001
	Negative	16	42.1	13	34.2	9	23.7	38	100		
	Total	32	21.6	40	27	76	51.4	148	100		
Dyslipidemia	Positive	26	23.6	39	35.5	45	40.9	110	100	21.16	0.001
	Negative	6	15.8	1	2.6	31	81.6	38	100		
	Total	32	21.6	40	27	76	51.4	148	100		
Priapism	Positive	0	0	0	0	22	100	22	100	24.481	0.001
	Negative	32	25.4	40	31.7	54	42.9	126	100		
	Total	32	21.6	40	27	76	51.4	148	100		
Peyronie	Positive	16	37.2	9	20.9	18	41.9	43	100	8.708	0.013
	Negative	16	15.2	31	29.5	58	55.2	105	100		
	Total	32	21.6	40	27	76	51.4	148	100		

**Table 3:** Distribution of the groups in terms of Doppler ultrasonography results.

										Chi-Square Test	
		Group I		Group II		Group III		Total			
		*n*	%	*n*	%	*n*	%	*n*	%	Chi-Square	*p*
PDDU	Arterial insufficiency	20	30.3	25	37.9	21	31.8	66	100	56.605	0.001
	Venous insufficiency	3	14.3	0	0	18	85.7	21	100		
	Arterial/Venous insufficiency	9	30	14	46.7	7	23.3	30	100		
	Normal	0	0	1	3.2	30	96.8	31	100		
	Total	32	21.6	40	27	76	51.4	148	100		
PSV	30 cm/s>	3	5.6	1	1.9	50	92.6	54	100	58.248	0.001
	30 cm/s<	29	30.9	39	41.5	26	27.7	94	100		
	Total	32	21.6	40	27	76	51.4	148	100		
EDV	8 cm/s>	12	19	14	22.2	37	58.7	63	100	2.436	0.296
	8 cm/s<	20	23.5	26	30.6	39	45.9	85	100		
	Total	32	21.6	40	27	76	51.4	148	100		
RI	0.8>	14	19.7	11	15.5	46	64.8	71	100	11.744	0.003
	0.8<	18	23.4	29	37.7	30	39	77	100		
	Total	32	21.6	40	27	76	51.4	148	100		

PDDU: Penile duplex Doppler ultrasound, PSV: Peak systolic velocity, EDV: End diastolic velocity, RI: Resistance index.
